# Dynamics and Kinetics of Cupric Ion Removal from Wastewaters by Tunisian Solid Crude Olive-Oil Waste

**DOI:** 10.3390/ma12030365

**Published:** 2019-01-24

**Authors:** Besma Khiari, Manel Wakkel, Souhir Abdelmoumen, Mejdi Jeguirim

**Affiliations:** 1 National School of Engineers of Carthage, 45 rue des Entrepreneurs, Tunis 1002, Tunisia; besmakhiari@yahoo.com; 2 Laboratoire des Sciences et Technologies de l’Environnement; BP 50 Borj Cedria Technopark 2050 Hammam-Lif; manel.elwakkel@gmail.com; 3 National Institute of Applied Science and Technology (INSAT), University of Carthage, Tunis 1080, Tunisia; souhir.abdelmoumen76@gmail.com; 4 Université de Strasbourg, Université de Haute-Alsace, Institut de Science des Matériaux de Mulhouse (IS2M), UMR 7361 CNRS-UHA, 15 rue Jean Starcky, BP 2488, 68057 Mulhouse CEDEX, France

**Keywords:** copper, olive mill solid waste, equilibrium, mass transfer, modeling

## Abstract

The present paper aims to develop a low cost, efficient, and environmentally-friendly process to purify (industrial) waters contaminated by copper by the use of oil mill wastes, through kinetic, thermodynamic, and equilibrium investigations. To do so, the raw adsorbent was characterized using different analytical techniques including X-ray diffraction (XRD) and Fourier transform infrared (FTIR) spectroscopy. Then, the interaction between copper and olive residues were examined during batch adsorption tests at various operating parameters, such as pH, initial concentration, contact time, and particle size. Kinetic data were best fitted with Broeurs-Sotolongo kinetic model. Additionally, it was found that film and intraparticle diffusion steps controlled simultaneously the mass transfer of copper onto olive mill solid waste. Among the eight tested models, Broeurs-Sotolongo isotherm suited the most the sorption, with regards to the function errors analysis. It was deduced that the adsorption of copper does not involve chemical bonds with high energy which allows easier regeneration steps and higher number of biosorbent regeneration cycles without any need for applying high temperature in the desorption reaction systems. The adsorption capacity (18.93 mg/g) calculated on the basis of this model was close to the experimental value (18.4 mg/g) but more interestingly it brought up that 50% of the generated amounts of olive wastes in Tunisia could eliminate 1.84 kTons of copper from industrial waters.

## 1. Introduction

The rapid development of industries threats aquatic and human lives through the discharge of contaminated wastewater on the environment [1,2]. Metal ions are the major water pollutants, with 300–400 MT annually [3], causing great danger to aquatic life and human health [4,5]. Copper is one major heavy metal used in several industrial processes such as textile, mining, wire drawing, paint manufacturing, electronics, electroplating, petrochemical, and printing operations [6,7,8]. The discharge of wastewater rich in copper in the environment can affect significantly plants, soil, atmosphere, animals, and human life upon entering the food chain [4,9]. It, therefore, became urgent to treat contaminated wastewaters before their discharge to the environment [10]. 

Different water treatment technologies are generally employed for heavy metals removal including coagulation, ion exchange, membrane process, reverse osmosis, solvent extraction, chemical precipitation and electroflotation [3,11,12]. The high operating and equipment costs, the extensive use of chemicals and the relatively low efficiency of these methods, especially in low heavy metals concentration (<100 mg.dm^−3^) limit their application at large scale in industrial wastewater treatment plants [11]. In contrast, being simple, economic, eco-friendly and low-energy requiring technology, adsorption processes have been proved to be successful to remove heavy metals from aqueous effluents [13].

In order to face the high costs of activated carbon, attention turned to cheap, abundant, and renewable adsorbents [11,14,15]. A large number of agricultural and industrial solid wastes were utilized to remove heavy metals from water such as, banana peels [16,17], tea factory waste [18], date stones [19,20], potato peels [21], waste apple pulp in cider production [22], rice husks [7], pistachio-nut shells [23], and coffee wastes [24]. In these works, the maximum adsorption capacity of copper ranged between 10 and 84 mg/g according to the waste and to the operating conditions of the adsorption process.

In addition to these by-products, olive-waste cakes (generated from olive oil industry) received renewed attention since they are generated in huge quantities especially in Mediterranean countries. In Tunisia, which is the first producer of olive oil in the world, around 2 × 10^5^ tons of olive residues are produced annually [4,25,26] and could be used as efficient adsorbents [27,28]. In fact, it has been reported that solid olive wastes can be converted to adsorbent materials with a cost less than $50/ton versus $4500/ton for activated carbon [4,29].

Despite the high number of studies dealing with heavy metals removal by olives residues, mechanistic studies are still not completely illustrated [4]. For instance, Veglio et al. conducted a microanalysis using a scanning electron microscope equipped with an energy dispersive X-ray analyzer to collect qualitative information on the elemental composition of olive pomace. The photographs showed that olive cake was quite porous media, with a pore distribution characterized mainly by mesopores (2 nm–50 nm) [30]. Infrared spectroscopy analysis by Pagnanelli et al. showed the presence of carboxylic groups (elongation of the C=O bond at 1704 and 1643 cm^−1^) and phenolic groups (elongation of the C=C bond at 1507 cm^−1^). These are thought to be the active sites, which are the main responsible for the retention of heavy metals. The profile of pH against poured NaOH volume suggested also numerous and various weakly acidic active sites [29]. In the same study, authors found that Cu^2+^ retention was the fastest (2h) and with a rate up to 85%, followed by Hg^2+^ (63%; 4h), Pb^2+^ (62.5%; 6h) and Zn^2+^ (16%; 3h). 

Several and simultaneous mechanisms involved in the retention of cupric ions on olive cakes were evoked in literature and, the demonstration of a particular mechanism remains particularly difficult and not usually soundly-based [30,31]. For example, the fact that the adsorbent efficiency decreased in acid media (pH < 5), let the authors speculate with caution an exchange of ions between the moving protons of the acid groups and Cu^2+^ in solution [32]. However, for pH > 5, copper is still precipitated as active sites are probably in molecular (non-ionized) form [29].

Contact times ranging from one minute to five days have been cited in the literature. However, the maximum adsorption yields are generally reached after a short contact time, around one hour [33]. 

The particle size has also an influence on the adsorptive properties of olive oil mill residues: the finer, the more efficient. For example, the surplus was about 45% for grain diameter from 355 down to 212 μm in Pagnanelli et al. [29]. 

As for isotherms, conventional two-parameter models were applied in literature to cupric ions removal from olive cake. Langmuir model was the most fitting one such as in the studies carried out by Pagnanelli et al. [34]. Studies that have used three-parameter models in biosorption are rare. Moreover, and as far as we know, fractal models such as Brouers-Sotolongo or Khan have never been tested with the present adsorbent-adsorbate. 

In kinetic studies, the model usually adopted is that of pseudo-second order, as it has proved to generally fit the sorption by the lignocellulosic matters. As an example, one can cite the kinetics established by Alslaibi et al. concerning Cu^2+^ adsorption by activated carbon prepared from olive stone waste [35]. However, no academic work investigating the fitting with other kinetic models, such as Elovitch or Brouers-Sotolongo, has been found in the literature review.

The aim of the present study is then to explore the use of local, abundant, low-cost, and renewable Tunisian olive mill solid waste to remove copper (II) ions from aqueous solution through rigorous kinetic, dynamic and equilibrium studies. The novelty of this work consists among others in the use of different equilibrium models with three parameters which, although being more accurate, are still not applied in biosorption studies. Additionally, rarely-used but more rigorous kinetic models are applied to this system in order to implement the process at industrial scale. Finally, and contrary to the most reported results, the kinetic and the equilibrium model parameters were calculated by non-linear fitting methods. This may help the determination of more appropriate operating conditions that may suit biomaterials from the Mediterranean countries, in general, and from Tunisia, in particular. 

## 2. Materials and Methods 

### 2.1. Adsorbate Solution Preparation

A solution of copper (1000 mg/L) was prepared by dissolving CuSO_4_.5H_2_O salt in deionized water. According to preliminary tests, the initial pH of the stock solution was adjusted to 5.25. This value is the optimum pH for copper solubility. The cupric ions concentration was measured by flame atomic absorption using an AAS VARIO 6 spectrometer (Analytik Jena, Jena, Germany). The flame is generated by an air/acetylene mixture and the wavelength used to measure copper concentration was fixed at 324 nm. 

### 2.2. Olive Mill Solid Residues Preparation

The material used in this study was olive pomaces obtained from a local artisanal olive mill in Ksour Essef (Governorate of Mahdia) in the center of Tunisia. They are made of crushed stones and olive pulp (flesh and skins). The samples were washed several times with distilled water to remove dust, filtered under vacuum, dried in an oven at 70 °C during 24 h and then crashed and sieved to obtain different particle sizes ranging from 0.5 to 2.8 mm. 

### 2.3. Adsorbent Characterization

#### 2.3.1. Proximate Analysis

The water content of olive mill solid waste was determined by drying the olive pomace at 105 °C and the moisture percent was calculated when a constant mass was reached. The ash content was obtained by heating a quantity of olive pomace in a muffle furnace (BTI, Maharashtra, India) at 550 °C during 15 min [36].

Lipid levels were determined with the help of the Soxhlet extraction procedure, during which a mass of 2 g of olive pomace was introduced in a packet made of filter paper. Hexane was used to extract oil during four hours and was then evaporated to recover the oil fraction.

#### 2.3.2. Textural Properties

The apparent surface (S_BET_) and pore volume were determined by applying the Brunauer, Emmett, and Teller (BET) method [37,38] during nitrogen (N_2_) adsorption analysis. The sample was firstly degassed under vacuum in order to evacuate gas and H_2_O molecules settled onto the sample. Then, the sample holder was immersed in a liquid nitrogen bath. A Micrometrics ASAP 2000 analyzer (ASAP, GA, USA) was used to determine the nitrogen adsorption isotherms at 77 K for P/P_0_ ranging from 0.05 to 0.25. This range of relative pressure allows to avoid the overestimation or the undervaluation of the specific surface area [6].

#### 2.3.3. XRD Analysis

The crystallographic structure of olive mill solid waste was studied by carrying an X-ray diffraction (XRD) analysis. A mass of 0.5 g of ground olive pomace was firstly placed between the blade and the slide and then introduced into the diffractometer. X-ray patterns were recorded with a PHILIPS PW 3040 diffractometer (Philips, Westhorst, Netherland). The detector angle (2θ) ranged between 0 and 70° and the wavelength of X-radiation was adjusted to 1.789 A° [4].

#### 2.3.4. FTIR Analysis 

The chemical properties of olive pomace were highlighted by a Fourier transform infrared (FTIR) spectroscopy analysis. It allows identifying the functional groups present on the adsorbent surface and involved in the adsorption of heavy metals. A few milligrams of olive pomace (1.5 mg) were mixed with 250 mg of potassium bromide (KBr, Merck) and then pelletized by applying a pressure of 10 atm during 10 min. The pellet obtained was introduced into the FTIR spectrophotometer (Perkin-Elmer Spectrum 2000) (Perken-Elmer, Dresden, Germany) and the FTIR spectrum was recorded between 4000 and 400 cm^−1^ which corresponds to the middle region of the infrared portion of the electromagnetic spectrum [6].

### 2.4. Adsorption Tests 

All sets of adsorption experiments were carried out in batch systems where 1 g of olive pomace was added to 100 mL of copper solution. The initial pH was adjusted by using hydrochloric acid HCl (0.1N) or sodium hydroxide NaOH (0.1N) solutions. The mixture was agitated at 180 rpm in a thermostatic shaker kept at 20 °C. At the end of experiments, solid phase was separated from metal solution by centrifuging the mixture at 180 rpm during 15 min. The residual concentrations as well as the initial concentration of copper were measured by atomic absorption spectrophotometer (Shimadzu, Kyoto, Japan). The removal percent of copper was calculated according to the following equation:

(1) $$\%\;\mathrm{Removal}=\frac{\left(C_{i}-C_{e}\right)}{C_{i}}\cdot 100$$

where C_i_ and C_e_ are the initial and final concentrations of copper (mg/L).

Experimental conditions including the range of pH, initial concentration, contact time, and particle size are reported in Table 1. All tests have been carried out three times.

### 2.5. Kinetic and Equilibrium Batch Studies

In order to characterize the adsorption capacity and the mass transfer mechanisms of copper inside olive mill solid waste particles, equilibrium and kinetic studies were conducted. 

Adsorption isotherms were established by immerging 1 g of olive pomace in aqueous solutions with different copper concentrations varying from 20 to 320 mg/L. Preliminary adsorption experiments were performed to determine the optimum pH value for maximum adsorption. Once the pH solution was adjusted, reactional mixture was stirred for 180 min at 20 °C. The solution was then separated from the adsorbent after a centrifugation step at 180 rpm for 15 min. 

The quantity of cupric ions adsorbed onto a unit mass of olive pomace (q_e_) was calculated according to the following equation:

(2) $$q_{e}=\frac{\left(C_{i}-C_{e}\right)V}{w}$$

where q_e_ is the adsorbent phase concentration after equilibrium (mg Cu^2+^ ion/g adsorbent), V is the solution volume (L), and w is the dry weight of olive mill solid waste (g).

As for thekinetic experiments, they were carried out by fixing the concentration of copper to 200 mg/L and varying the contact time from 30 to 300 min. At the different intervals of time, samples were extracted from the mixture and separated from the solid phase. 

In order to assess the best fitted equilibrium and kinetic models, an error analysis was carried out with the help of four error functions summarized in Table 2: The sum of the squares of the errors (SSE), Hybrid fractional error function (HYBRID), Marquardt’s percent standard deviation (MPSD), and correlation coefficient (R^2^) [39].

#### 2.5.1. Kinetic Models

Pseudo-first order, pseudo-second order, Elovich, and Brouers-Sotolongo models were applied to describe the mechanisms of pollutants removal by adsorption process. The limiting steps in mass transfer of ions/molecules to the adsorbent phase can be determined by applying film and intra-particle diffusion models. All of these models are presented in Table 3. 

#### 2.5.2. Equilibrium Models

Equilibrium data is generally useful to describe the pollutants distribution between solid and fluid phases at a constant temperature. In the present study, four two-parameter (Langmuir, Freundlich, Temkin, and Dubinin- Radushkevich) and five three-parameter (Brouers-Sotolongo, Khan, Hill, Toth, and Kobble-Corrigan) adsorption isotherms were applied to experimental data. The corresponding equations are seen in Table 4.

## 3. Results and Discussions

### 3.1. Adsorbent Characterization

In order to identify the mechanism of copper removal from aqueous solution, the characterization of the biosorbent is an important first step. To do so, physical (moisture content, XRD, N_2_ adsorption) and chemical properties (FTIR analysis) of olive mill solid residues were studied. 

#### 3.1.1. Moisture, Oil, and Ash Content

The water content of the pre-dried olive residues was 10.25%, which is similar to the 10.4% recorded by Gonzales-Garcia (2018) [6]. These values are comparable to many other bio-sorbents such as orange peels (9.2%), Pecan shells (10.4%), peach stones (9.3%), stone pines (9.8%) [6]. The ash rate was 3.42% and oil content 11.4%. The low value of ashes can be explained by the organic nature of the olive mill solid waste whereas the high fat percentage could give information about the chemical groups present on the olive residues such as triglycerides. Ester and carboxyl groups can be considered as the abundant functional groups on the olive pomace.

#### 3.1.2. XRD analysis

**Figure 1:**
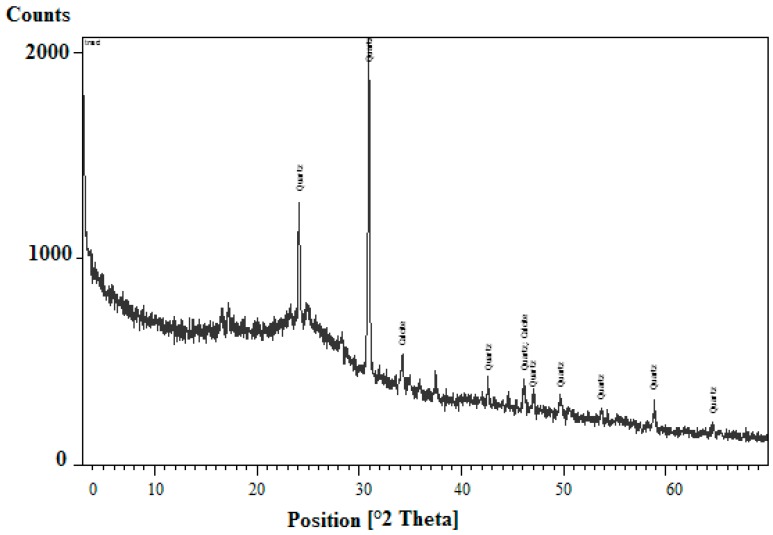
X-ray diffractogram of olive mill solid waste.

X-ray diffraction (XRD) analysis is generally released to assess the chemical structure of olive mill wastes and informs whether the material is amorphous or crystalline [4]. The X-ray diffractogram of the olive residues is shown in Figure 1.

One can see that the shape of the XRD diagram provides an idea about the order/disorder degree of the structure of the adsorbent whereas the peaks indicate the presence of calcite CaCO_3_ and quartz SiO_2_ as main crystallized compounds constituting the olive residues. It is worth mentioning here that calcite, which is crystallized in the rhombohedral structure, is one of the most abundant carbonates in nature. 

#### 3.1.3. BET Analysis

With the BET method, olive mill solid waste presented a very small specific surface which did not exceed 2 m^2^/g but which is still in the same order of magnitude of the surface area (5.3 m^2^/g) of bio-chars produced from olive mill solid waste [26]. Additionally, the pore volume of the olive pomace was estimated to be 0.0128 cm^3^/g. Similarly low values were found in previous studies [26]. 

#### 3.1.4. FTIR analysis

**Figure 2:**
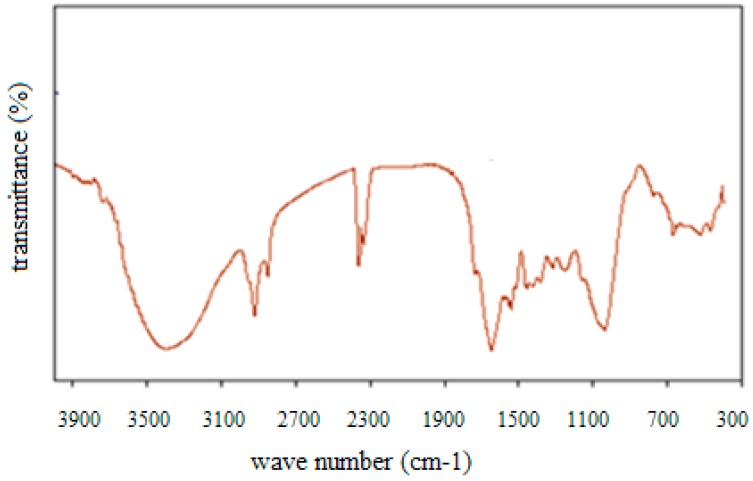
FTIR of olive mill solid waste.

Fourier transform infrared spectrum of olive pomace revealed the presence of peaks with different wavelengths corresponding to different functional groups (Figure 2). 

A broad band of the O–H stretching vibration appears at a wavelength of 3420 cm^−1^. It corresponds to both free and bonded hydroxyl groups which are generally characteristics of alcohols, carboxylic acids and phenols. In fact, olive mill solid waste is a solid material, constituted by cellulose, hemicelluloses and lignin. Oxygen functional groups such as hydroxyl, ether and carbonyl are the main chemical groups of cellulose and hemicellulose molecules [4,40,41,42]. The peak around 1704 cm^−1^ is attributed to the carbonyl group (C=O) which is often present on the hemicelluloses and lignin molecules [43]. Additionally, olive pomace contains C–H stretching vibration around 2900 cm^−1^ which indicates the presence of an alkene functional group [44]. There are also some peaks in the fingerprint region between 1500 and 400 cm^−1^. In fact, the FTIR band around 1000 cm^−1^ corresponds to C–O–C groups generally found in cellulose molecules [41]. 

All these functional groups on the surface of olive mill solid waste would allow interactions between cupric ions and adsorbent surface. Thus, electrostatic interactions and/or complexation can be proposed as possible mechanisms of copper removal from aqueous solution. Similar findings were highlighted by Hawari et al. who studied the removal of cupric ions by oil mill solid residues (OMSR). According to FTIR analysis before and after adsorption, it was concluded that OMSR may involve different mechanisms as ion exchange, electrostatic attraction and complexation [41]. 

### 3.2. Impact of Operating Conditions on Copper Adsorption

#### 3.2.1. Contact Time Effect

**Figure 3:**
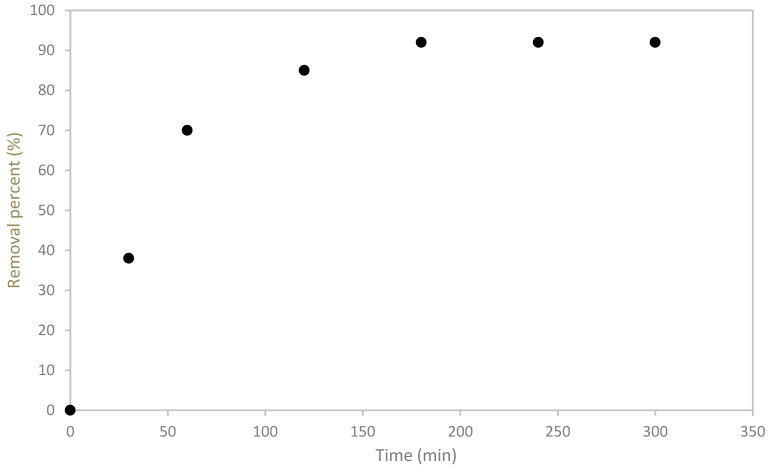
The effect of contact time on the removal of copper by olive mill solid waste.

The Cu(II) removal versus contact time is shown in Figure 3. One can see that the adsorption rate was rapid: within the initial 60 min. Beyond, the removal process slows down, reaching a constant value around 92%.

In fact, 70% of copper was removed during a contact time around 60 min. Then, the removal rate of copper decreased for a contact time comprised between 60 and 180 min. For longer residence times, the adsorbent becomes saturated and no enhancement of the sorption capacity is recorded. The fast adsorption rate can be attributed to the availability of active sites at the beginning, as well as to the functional groups present on the surface [45]. Thus, the resistance of mass transfer was low in the first stage of the process and increased with the contact time, which led to the sorption rate decrease.

#### 3.2.2. Particle Size Effect 

**Figure 4:**
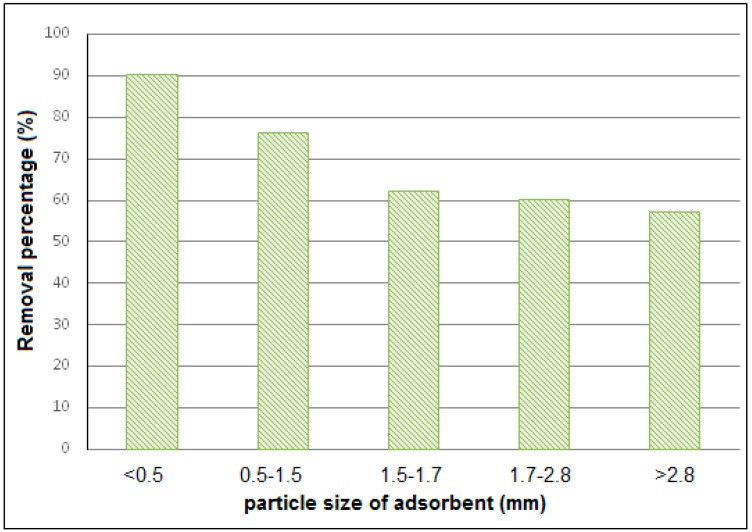
The removal yield of copper from aqueous solution by olive pomace.

In order to assess the particle size influence on the adsorptive capacity of olive cakes, five ranges of particle sizes (from 0.5 to 2.8 mm) were used (Figure 4).

According to Figure 4, the retention of cupric ions was decreased by about 33% between fine powder smaller than 0.5 mm and particles with a diameter superior to 2.8 mm. In fact, the smaller are the particle sizes, the greater are the rates of diffusion and of adsorption. This finding was highlighted in several previous studies which indicated that the increase of particle size leads to a decrease in the rate of adsorption since the internal mass transfer zone becomes larger, which reduces the intra-particle diffusion [36]. 

In addition, it should be mentioned that the size of particles affects not only the kinetic of the adsorption process but also the capacity of the adsorbent to remove pollutants. In fact, the reduction of the size of particles enhances the specific surface and, thus, the quantity of the retained adsorbate and the efficiency of the adsorption process.

#### 3.2.3. pH Effect

**Figure 5:**
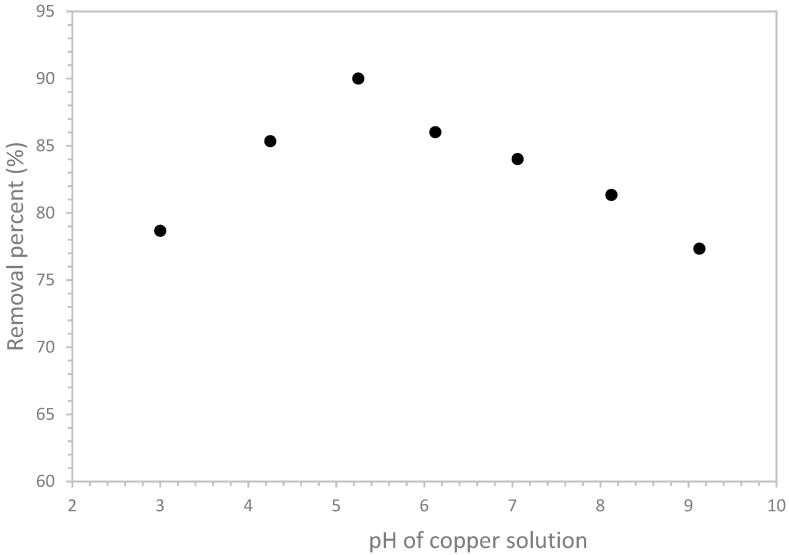
Effect of pH on copper removal from aqueous solution by olive oil pomace.

The pH solution is one of the most significant factors affecting the adsorption process since it controls the surface charge density of the adsorbent and the metal species. The effect of pH solution on the removal of the cupric ions from aqueous solution by olive mill solid residues is shown in Figure 5.

The removal yield of copper rose with the increase of pH from 3 to 5.25 and reached a maximum at pH equal to 5.25. After that, the amount of copper adsorbed decreased when the pH values moved from 5.25 to 9.125.

The high removal percent (90%) of copper at acidic pH (5.25) can be explained by the high solubility of the divalent ionic form of copper (Cu^2+^) at this pH. Similar results were highlighted by Hawari et al. who found that the high adsorption capacity of copper by olive mill solid residues was obtained at pH 5 [41]. 

At low pH values (<5.25), and due to the high H^+^ concentration, the competition between H^+^ and Cu^2+^ ion species for the sites of adsorbent slows down the adsorption. However, at pH values higher than the optimum value (5.25), Cu^2+^ ions tend to precipitate out of solution as hydroxides (Cu(OH)_2_) according to the following reaction [46]:

(3) $${\mathrm{Cu}}^{2+}+2{\mathrm{OH}}^{-}↔\mathrm{Cu}{\left(\mathrm{OH}\right)}_{2}$$

The solubility constant K_sp_ of copper hydroxide (Cu (OH)_2_) can be written as follows:

(4) $$K_{\mathrm{sp}}=\left[\mathrm{Cu}\right]{\left[\mathrm{OH}\right]}^{2}$$

In fact, most heavy metal hydroxides are relatively insoluble at certain pH [47]. The pH which allows the formation of the hydroxide deposit was calculated according to Equation (5) and it was found equal to 5.59.

(5) $$\mathrm{pH}=14+0.5\log K_{\mathrm{sp}}-0.5\;\log\left[\mathrm{Cu}\right]$$

It should be mentioned that the removal yield of copper is still not negligible at high values of pH solution (around 77.33%). Similar finding was highlighted by Hawari et al. [41].

In addition, it should be mentioned that pH influences not only the chemical properties of copper in aqueous solution but also the chemical properties of the functional groups of the oil mill solid waste. 

Indeed, oxygen functional groups (hydroxyl, ether, and carbonyl) revealed by FTIR are to be correlated with the maximum removal rate obtained at pH 5.25. At a pH < 6, acid groups at the surface are more likely to be involved. This also would explain the pH-dependence observed. In fact, at this value, olive residues have a negative charge due to the excess of negatively-charged functional groups. This negative charge is probably due to the pKa of hydroxyl (10) and of phenol (22), above the value of the optimum pH (5.25). As for carbonyl, the pKa (4) is below to the optimum pH. Thus, this metal removal (depending on pH) is itself dependent on the ligands responsible for the uptake of copper, for instance carboxyl and phenol functional groups. 

On the issue of these results, it can be concluded that the removal efficiency of cupric ions depends strongly on pH solution which indicates that electrostatic interactions and/or ion exchange are possible mechanisms of copper biosorption [6,41]. 

#### 3.2.4. Initial Concentration Effect

**Figure 6:**
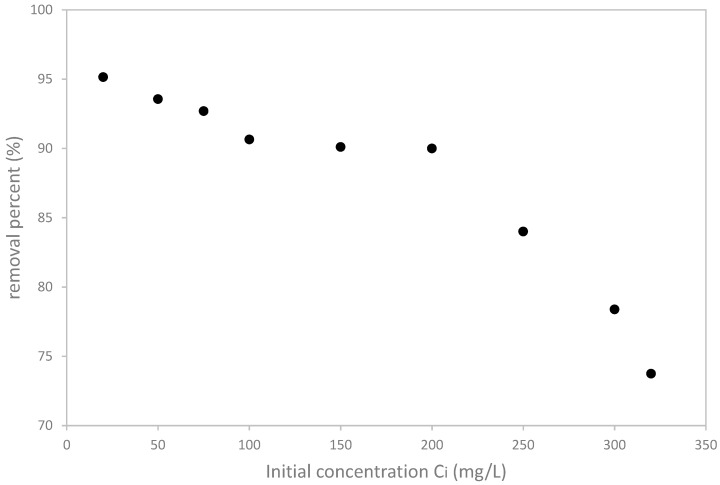
Effect of the initial concentration of copper on its removal yield by olive pomace.

The influence of the initial concentration of copper on the adsorption process is shown in Figure 6.

It is clear that the increase of initial concentration of copper leads to a decrease in the copper removal efficiency. In fact, around 21% of the removal capacity was lost when copper concentration increased from 20 to 320 mg/L. This result can be explained by the saturation of the adsorption sites which is due to the presence of a high quantity of cupric ions.

This result implies that only small fraction would be retained if Cu^2+^ concentration is high. Olive mill solid waste is was more efficient at low concentrations where the cupric ions are totally or predominantly retained and, if the Cu^2+^ concentration becomes very large, only a small fraction was retained. Nevertheless, it should be noticed that the concentrations of cupric ions in industrial wastewaters do generally not reach such the high values tested in the present study. As an example, the wastewaters of tanneries contain a concentration of cupric ions less than 30 mg/L which is in the same order of magnitude of the concentrations used in this work and for which where a high removal yield was recorded.

### 3.3. Kinetic Study

**Figure 7:**
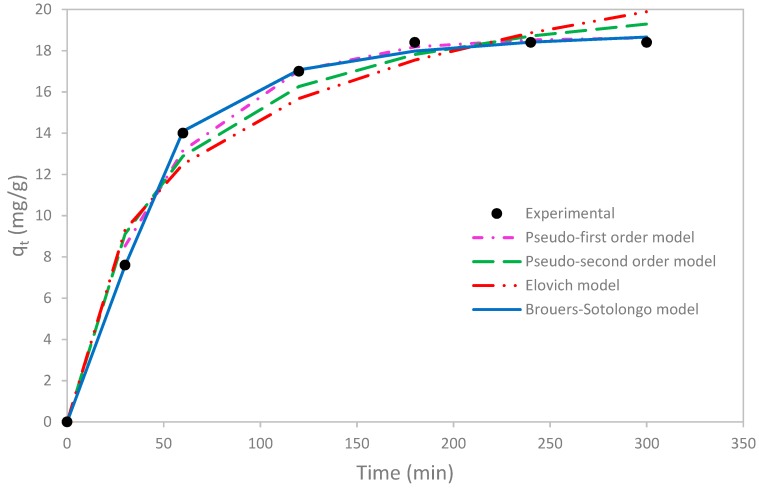
Experimental and theoretical kinetic data of copper adsorption on olive mill solid waste.

Several kinetic models were applied namely pseudo-first order, pseudo-second order, Elovich, and Brouers-Sotolongo models. Model parameters were determined by the non-linear fitting method and experimental, as well as fitted curves were presented in Figure 7.

According to Figure 7, it is clear that the kinetic uptake of copper was faster during the first 60 min and became constant after a contact time of 180 min (q_t_ = 18.4 mg/g). This last same value was also reached by Tchoumou et al. [45]. 

The fast sorption in the first stage can be explained by the abundance of the functional sites. This is advantageous at industrial scale since it allows treating heavy-metal contaminated water with a minimum of contact time and with lower operation costs. Afterwards, adsorption sites become saturated and the sorption rate decreases. 

With such capacity and given the average produced and non-recovered quantity of olive cake in Tunisia, around 1.84 kTons of copper could be removed from contaminated waters. This last quantity, being more important than that actually produced by the Tunisian industries, possible import routes to neighboring countries could be envisaged. 

In order to assess the best fitted kinetic model, four error functions (SSE, HYBRID, MPSD, and R^2^) were calculated. 

According to the results reported in Table 5, Brouers Sotolongo is the best fitting model since the correlation coefficient R^2^ (0.998) exhibited the highest value and SSE (0.198), HYBRID (0.011) and MPSD (0.0006) the lowest. Besides, the adsorption capacity (18.93 mg/g) calculated on the basis of this model was the closest to the experimental value (18.4 mg/g).

Using the parameters of the Broeurs-Sotolongo model (γ, n and τ), the half sorption time (τ_50%_) can be determined according to the following equation [48,49]:

(6) $$\tau_{50\%}=\tau {\left(\frac{2^{\left(n-1\right)}-\left(n-1\right)}{\left(n-1\right)}\right)}^{1/\gamma }$$

The half sorption time is as the time at which the half of the adsorbate has been removed. This parameter was found to be 31.56 min in this study. This result is in adequacy with the experimental output since the first step of the sorption reaction occurs within 60 min, during which 76% of copper was removed.

The comparison of the pseudo-first and the pseudo second order models, as the most used kinetic models, showed that the latter model fit less the kinetic uptake of copper compared to the former. This indicates that the adsorption kinetic was not governed by chemical mechanisms. 

For Bohli et al., the pseudo-first order model also gave quite good prediction with R^2^ = 0.970 (q_e,cal_ = 0.145 mmol/g) but the pseudo-second order (q_e,cal_ = 0.139 mmol/g) was more suitable (R^2^ = 0.999) for copper removal by activated carbon issued from olive stone waste [50].

On other side, Elovich prediction was the worst with the highest SSE, HYBRID and MPSD and the lowest R^2^. According to the assumptions of this model, it can be deduced that desorption reactioncan occur and the dominant adsorption mechanism cannot be chemical sorption. This finding strengthened the conclusion highlighted through the analysis of the poor fitting of the pseudo-second order model to the experimental data.

The four kinetic models cited above inform us about the sorption rate but they did not give information about the adsorption mechanisms and the controlling step of the copper adsorption by olive mill waste. For this purpose, film and intraparticle diffusion models were applied to estimate which step controls more the overall adsorption rate. Intraparticle (K_in_, C) and film (K_f_, D_f_) diffusion model parameters were determined by the non-linear method using the solver add-in facility for Microsoft Excel (USA) (and were reported in Table 6.

The effective diffusion coefficient of the copper onto olive residues (D) can be calculated from the intraparticle rate sorption (K_in_) by using the following equation:

(7) $$K_{\mathrm{in}}=6\frac{q_{e}}{r}\sqrt{\frac{D}{\pi }}$$

where D is in (cm^2^/s) and r is the mean radius of adsorbent particles (cm).

The free diffusivity D_0_ (m^2^/s) of the copper in diluted solutions can be determined according to Stokes-Einstein equation:

(8) $$D_{0}=\frac{K_{B}\cdot T}{6\cdot \pi \cdot \eta \cdot r}$$

where:

K_B_: Boltzmann constant (J/mol)
T: Temperature (K)
η: Dynamic viscosity (Pa.s)
r: Copper radius (m)

The calculated ratio D/D_0_ (10^−3^) indicates that the diffusivity of copper inside olive mill residues was reduced by a factor of 1000 compared to its diffusivity in diluted solutions. This finding indicates the high hindrance of copper by the adsorbent which is expected since the physical properties of olives residues were limited (BET surface, porosity).

At the same time, C parameter was equally determined and was estimated to be 5.89 mg/g. Since this parameter is different from zero, it can be deduced that the kinetic uptake of copper was not controlled by only intra-particle diffusion phenomenon and that other controlling mechanisms might be involved simultaneously. For this reason, a film diffusion model was tested and the corresponding parameters (K_f_ and D_f_) were determined and reported in the same Table 6. The obtained values let us conclude that film diffusion model was not the sole controlling step in copper adsorption reaction since the plot relating Bt to Ln(1-F) did not pass through the origin (data not shown). This finding suggests that another mass transfer mechanism controls the sorption uptake which is probably the intraparticle diffusion step. 

In order to determine the dominating diffusion mechanisms, the Biot number (Bi) was calculated according to the following equation:

(9) $$\mathrm{Bi}=\frac{k_{f}\cdot d\cdot C_{0}}{2\cdot \rho_{p}\cdot D\cdot q_{e}}$$

where k_f_ (cm/s) is the film diffusion constant, D (cm^2^/s) the intraparticle diffusion coefficient, C_0_ (mg/L) the initial liquid-phase concentration, d (cm) the mean particle diameter; and ρ_p_ (g/cm^3^) the adsorbent density and q_e_ (mg/g) is the solid phase concentration at equilibrium. 

The adsorbent density used to calculate the Biot number was adopted from a previous work of Ferhat, et al. [51].

In previous works, three intervals of Biot number (Bi) were adopted, as follows:

■ If Bi ≪ 1, film diffusion is the controlling step;
■ If Bi ≫ 100, intra-particle diffusion is the controlling step; and
■ If 1 < Bi < 100, Film and intraparticle diffusion are the limiting steps.

Again, and given the Biot number comprising between 1 and 100 for the current system, film and intra-particle diffusion mechanisms control simultaneously the adsorption process of copper by olive mill solid wastes. However, given the low porosity, intraparticle diffusion would not be dominant.

Thus far, these results suggest that faster agitation of the system and the enhancement of the physical (specific surface, porosity) and chemical (functional groups) properties of the adsorbent would allow improving the overall sorption rate.

### 3.4. Equilibrium Study 

**Figure 8:**
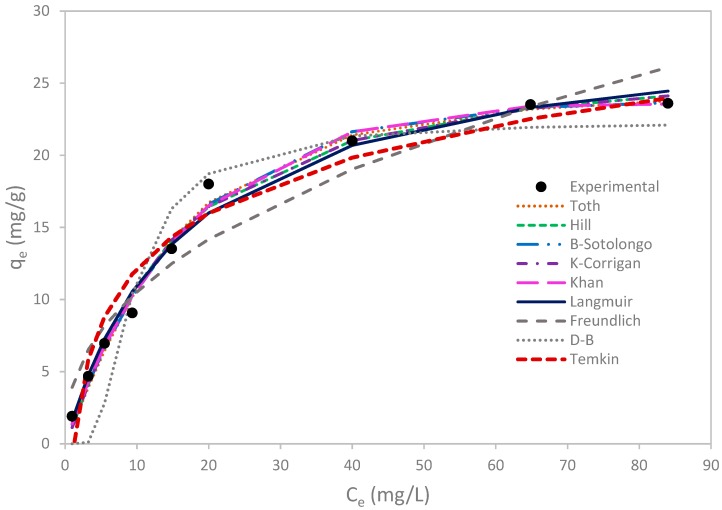
Experimental and theoretical isotherms of copper removal by olive mill solid residues.

In order to assess the affinity of the adsorbate towards the adsorbent, adsorption isotherm is usually established [52]. In this study, several models were applied and the results are reported (Table 7 and Figure 8).

The experimental adsorption capacity of copper by olive mill solid residues was q_max_ = 23.6 mg/g. This value indicates the high potential of this bio sorbent to remove cupric ions from aqueous solution. This value was compared to other biosorbents reported in previous studies dealing with copper removal from aqueous solutions and was found higher than that of the majority of the tested biosorbents (Table 8).

Moreover, as shown in Figure 8, experimental isotherm curve has the ‘’L‘’ shape which indicates that the uptake of cupric ions by olive mill residues was favorable and that the competition between solvent molecules and copper is weak.

In order to determine the most appropriate model to describe copper adsorption by olive mill solid residues, an error analysis was conducted by calculating R^2^, SSE, HYBRID, and MPSD values as indicated earlier. Thus, firstly, two-parameter models were compared with each other; then the two sets of models (two and three-parameter models) were compared by analyzing their corresponding error values (Table 7).

The comparison of the two-parameter models showed that Langmuir matched the best, with the highest R^2^ and the lowest SSE, HYBRID and MPSD, compared to Freundlich, Temkin, and Dubinin- Radushkevich model error values. Bohli et al. also found that Langmuir model described better the sorption of copper by activated carbon from olive stones than Freundlich model but also than Redlich-Peterson and Sips models with maximum calculated capacity q_max,cal_ = 17.15 mg/g and K_L_= 2.37 L/mg with R^2^ = 0.974 [50].

Langmuir suitability indicates that cupric ions are adsorbed by forming a monolayer of cupric ions around the olive waste particles, a conclusion reached also by Hawari et al. [41]. 

In order to improve the precision of models describing the adsorption of cupric ions, three-parameter models (Brouers-Sotolongo, Khan, Hill, Toth, and Kobble-Corrigan) were equally investigated and compared to two-parameter models. The analysis of error values led us to the conclusion that all the four three-parameter isotherms fitted better the equilibrium uptake of copper compared to two-parameter models, the lowest R^2^ value (0.989) being higher than that of Langmuir model (R^2^ = 0.987). Besides, and according to Table 7, Brouers-Sotolongo isotherm was found the nearest model to equilibrium data with lowest SSE (4.243), HHYBRID (0.529), and MPSD (0.128), and with the closest R^2^ to unity (R^2^ = 0.993). 

It should be mentioned that Khan and Toth models have the same correlation coefficient value (R^2^ = 0.992). However, the comparison of the SSE, HYBRID, and MPSD showed that Khan isotherm are more in adequacy with the experimental data compared to Toth model. Additionally, Hill and Koble-Corrigan have the same values of error functions and correlation coefficients. This similarity can be explained by the fact that both models have the same structure of mathematical equations.

## 4. Conclusions

In this paper, both solid waste management and used water management are proved likely to reduce the costs both in the oil mill industries and in activities that generate copper contaminated waters. Indeed, olive mill solid waste, cheap and abundant agro-industrial residue, was successfully applied to remove cupric ions from aqueous solutions.

The physical and chemical properties of olive pomace were investigated to bring out to which extent this waste can contribute in wastewater treatment by adsorption. Actually, XRD analysis highlighted the presence of crystalline matter (calcite, quartz) on the olive mill solid residues whereas FTIR helped detecting hydroxyl and phenol groups as the main chemical groups, which could help us speculate that copper could be efficiently removed by olive pomace, thanks to mechanisms such as electrostatic interactions.

An optimization study was conducted in order to understand the effect of different physical and chemical parameters (pH solution, initial concentration, particle size, and contact time) on the adsorption process. The solution initial pH value had a significant impact and, the optimum value was found equal to 5.25. The removal of Cu^2+^ decreased with the rise of its initial concentration due to the saturation of adsorption sites. As industrial effluents are rarely highly concentrated with copper, this would not be a brake for the development of the present process. The sorption kinetics was considered rapid since the adsorbate-adsorbent equilibrium is reached within 180 min. This is advantageous at industrial scale since it allows treating heavy-metal contaminated-water with minimum contact time and with lower operation costs. 

Finally, it was demonstrated that the maximum of copper adsorption was possible when adsorbent particle size was less than 0.5 mm. The more the solid is divided, the larger is the specific surface area and thus the greater is the adsorption capacity. The extra cost of the grinding operation is compensated by the efficiency of the process.

The kinetic study was carried out based upon the optimum experimental conditions determined earlier. The kinetic experimental data were fitted by four kinetic models (pseudo-first order, pseudo-second order, Brouers-Sotolongo, and Elovich). The Brouers-Sotolongo kinetic was the closest to experiments based on HYBRID, SSE, MPSD, and R^2^ values. These results confirm that the present biosorption does not involve chemical bonds with high energy and that desorption reactions can occur. The low energy of bonds allows easier regeneration steps and higher number of biosorbent regeneration cycles without any need to apply high temperature. Further calculations led to the conclusion that intraparticle and film diffusion models controlled simultaneously the mass transfer of copper inside olive mill solid waste particles. So far, this suggests that faster industrial agitation of the system and the enhancement of the physical (specific surface, porosity) and chemical (functional groups) properties of the adsorbent would allow improving the overall sorption rate.

Equilibrium data analysis is a necessary tool to evaluate the adsorption capacity of olive wastes and to judge the utility of the use of such process in industrial applications. Two and three-parameter isotherms were consequently applied and compared. Based on error analyses, the Brouers-Sotolongo isotherm was found to be the best-fitting model. Adsorption mechanisms were then elucidated and the limiting step determined, as the implementation of the process depends on their comprehension. Given the available amount of olive residues, up to 1.84 kTons of copper could be eliminated from wastewaters, placing crude olive-oil solid waste as a promising adsorbent but also as a powerful precursor for the manufacture of high efficient activated carbon in industrial effluent treatment processes. 

Finally, the adsorption capacity of commercial activated carbon CAC (priced 4500 US $ per ton) being around 100 mg/g is approximately four times greater than that of crude oil residue investigated in this study (23.6 mg/g) [57]. However, the cost of this latter after drying and grinding is around 50 US $ per ton that is 1/90 of CAC price. This rough calculation shows that the raw olive pomace is commercially viable and could replace efficiently CAC.

## Figures and Tables

**Table 1:** Experimental conditions.

	Contact Time	Particle Size	pH Solution	Initial Concentration
Contact time effect	30–300 min	0.5 mm	5.25	200 mg/L
Particle size effect	180 min	0.5–2.8 mm	5.25	200 mg/L
pH solution effect	180 min	0.5 mm	3–9	200 mg/L
Initial concentration effect	180 min	0.5 mm	5.25	20–320 mg/L

**Table 2:** Error functions.

Error Function	Equation
The sum of the squares of the errors (SSE)	$\overset{N}{\underset{i=1}{\sum }}{\left(q_{e,\exp}-q_{e,\mathrm{cal}}\right)}_{i}^{2}$
Hybrid fractional error function (HYBRID)	$\overset{N}{\underset{i=1}{\sum }}{\left[\frac{{\left(q_{e,\exp}-q_{e,\mathrm{cal}}\right)}^{2}}{q_{e,\exp}}\right]}_{i}$
Marquardt’s percent standard deviation (MPSD)	$\overset{N}{\underset{i=1}{\sum }}{{\left[\frac{\left(q_{e,\exp}-q_{e,\mathrm{cal}}\right)}{q_{e,\exp}}\right]}_{i}}^{2}$
Correlation coefficient (R^2^)	$\frac{\sum_{i=1}^{N}{\left(q_{e,\mathrm{cal}}-\bar{q_{e,\exp}}\right)}_{i}^{2}}{\sum_{i=1}^{N}{\left(q_{e,\mathrm{cal}}-\bar{q_{e,\exp}}\right)}_{i}^{2}+\sum_{i=1}^{N}{\left(q_{e,\mathrm{cal}}-q_{e,\exp}\right)}_{i}^{2}}$

q_e,exp_, q_e,cal_ and $\bar{q_{e,\exp}}$ express the experimental, mean experimental and the calculated values of adsorption uptake. N indicates observations number in the experimental data.

**Table 3:** Kinetic Models.

Model	Equation
Pseudo first-order	$q_{t}=q_{e}\left[\mathrm{Exp}\left({\mathrm{lnq}}_{e}-k_{1}t\right)\right]$
Pseudo second-order	$q_{t}=\frac{q_{e}^{2}k_{2}t}{q_{e}k_{2}t+1}$
Elovich	$q_{t}=\frac{1}{\beta }\ln\left(t\right)$
Brouers–Sotolongo	$q_{t}=q_{e}\left(1-{\left(1+\left(n-1\right){\left(\frac{t}{\tau }\right)}^{\gamma }\right)}^{\frac{-1}{n-1}}\right)$
Intra-particle diffusion	$q_{t}=k_{\mathrm{in}}t^{1/2}+C$
Film diffusion	$\ln\left(1-F\right)=-K_{f}\cdot t$
	$F=1-6/\pi^{2}\overset{\infty }{\underset{n=1}{\sum }}\frac{1}{n^{2}}\exp\left(-n^{2}\;\mathrm{Bt}\right)$
	$\mathrm{Bt}={\left(\sqrt{\pi }-\sqrt{\pi -\left(\frac{{F\pi }^{2}}{3}\right)}\right)}^{2}\mathrm{For}\;F<0.85$
	Bt=−0.498−ln(1−F)for F>0.85
	$B=\frac{\pi^{2}D_{f}}{r^{2}}$

**Table 4:** Equilibrium models.

Model	Equation
Langmuir	$q_{e}=\frac{q_{\max}\cdot K_{L}\cdot C_{e}}{1+K_{L}\cdot C_{e}}$
Freundlich	$q_{e}=K_{F}\cdot C_{e}^{1/n_{F}}$
Temkin	$q_{e}=\frac{\mathrm{RT}}{b_{T}}\ln\;\left(K_{T}\cdot C_{e}\right)$
Dubinin-Radushkevich	$q_{e}=q_{m}\;\mathrm{Exp}\;\left(-{\beta \varepsilon }^{2}\right)$
Koble–Corrigan	$q_{e}=\frac{A_{\mathrm{KC}}B_{\mathrm{KC}}C_{e}^{n_{\mathrm{KC}}}}{1+B_{\mathrm{KC}}C_{e}^{n_{\mathrm{KC}}}}$
Khan	$q_{e}=\frac{q_{K}b_{K}C_{e}}{{\left(1+b_{K}C_{e}\right)}^{a_{K}}}$
Hill	$q_{e}=\frac{q_{H}C_{e}^{n_{H}}}{K_{H}+C_{e}^{n_{H}}}$
Brouers–Sotolongo	$q_{e}=q_{\mathrm{BS}}\left(1-\exp\left(-K_{\mathrm{BS}}C_{e}^{\alpha_{\mathrm{bs}}}\right)\right)$
Toth	$q_{e}=\frac{q_{T}K_{T}C_{e}}{{\left(1+{\left(K_{T}C_{e}\right)}^{n_{T}}\right)}^{\frac{1}{n_{T}}}}$
Redlich–Peterson	$q_{e}=\frac{K_{\mathrm{RP}}C_{e}}{1+\alpha_{\mathrm{RP}}C_{e}^{\beta_{\mathrm{RP}}}}$

**Table 5:** Parameters of the kinetic models for the adsorption of copper by olive mill solid waste.

Model	Parameter	Value	SSE	HYBRID	MPSD	R^2^
Experimental q_e_ (mg/g)	18.4					
Pseudo-first order	K_1_ (min^−1^)	0.020	1.680	0.170	0.019	0.980
	q_e_ (mg/g)	18.664				
Pseudo-second order	K_2_ (mg/g.min)	0.001	5.290	0.487	0.051	0.928
	q_e_ (mg/g)	22.026				
Elovich	α (mg/g.min)	1.16	10.103	0.823	0.078	0.890
	β (g/mg)	0.218				
Brouers-Sotolongo	n	2.454	0.198	0.011	0.0006	0.998
	τ (min)	33.517				
	γ	2.430				
	q_e_ (mg/g)	18.933				

**Table 6:** Parameters of film and intraparticle diffusion models for copper uptake on olive pomace.

Intraparticle Diffusion Model					Film Diffusion Model		Bi
**K_in_ (mg min^0.5^/g)**	**C (mg/g)**	**D (cm^2^/s)**	**D_0_ (cm^2^/s)**	**D/D_0_**	**k_f_**	**D_f_ (cm^2^/s)**	20
0.83	5.89	1.74 × 10^−9^	1.76 × 10^−6^	10^−3^	1.87 × 10^−4^	1.18 × 10^−8^	

**Table 7:** Copper adsorption onto olive pomace at equilibrium and their fitting by two-parameter and three-parameter models.

Model		Parameter	Value	SSE	HYBRID	MPSD	R^2^
**Two-parameter models**	Langmuir	q_max_ (mg∙g^−1^)	29.285	7.341	0.568	0.066	0.987
		K_L_ (L∙mg^−1^)	0.060				
	Temkin	b_T_ (J∙mol^−1^)	435.640	26.651	5.879	2.297	0.952
		K_T_ (L∙mg^−1^)	0.889				
	Freundlich	K_F_ (L∙g^−1^)	3.965	36.156	4.563	1.391	0.928
		N	2.351				
	Dubinin- Radushkevich	q_m_ (mol∙g^−1^)	22.324	56.785	9.878	2.390	0.928
		β (mol^2^∙KJ^−2^)	1.13 × 10^−5^				
**Three-parameter models**	Brouers-Sotolongo	q_bs_ (mg/g)	23.797	4.243	0.529	0.128	0.993
		K_bs_ (L/mg)	0.059				
		A	1.003				
	Khan	q_K_ (mg/g)	65.325	4.690	0.493	0.108	0.992
		b_K_ (L/mg)	0.023				
		a_K_	1.561				
	Toth	q_T_ (mg/g)	24.875	4.846	0.676	0.185	0.992
		K_T_ (L/mg)	0.050				
		n_T_	1.738				
	Hill	q_H_ (mg/g)	26.936	6.074	0.786	0.217	0.989
		n_H_	1.184				
		K_H_ (L/g)	22.159				
	Kobbe-Corrigan	A_KC_ (L^nKC^ mg^1–nKC^/g)	26.936	6.074	0.786	0.217	0.989
		n_KC_	1.184				
		B_KC_ ((L/mg)^nKC^)	0.045				

**Table 8:** Comparison of rate constants for Cu(II) adsorption onto olive residues.

Adsorbent	q_max_ (mg/g)	Reference
Olive mill solid waste	23.6	This study
Sugarcane Bagasse	7.88	[45]
Orange peel	15.27	[53]
Peanut hull	21.25	[54]
Banana peel	27.78	[55]
Mango peel	46.09	[56]
Date palm waste	0.47	[20]

## Nomenclature

A	Arrhenius factor
A_1_	Constant (2.85 × 10^−5^ cm^2^g^1/3^/s.mol^1/3^)
a_K_	Khan model exponent
A_KC_	Koble–Corrigan parameter (L^nKC^ mg^1–nKC^/g)
B	Rate coefficient (s^−1^)
Bi	Biot number
b_K_	Khan model constant (L/mg)
B_KC_	Koble–Corrigan parameter (L/mg)^nKC^
b_T_	constant related to heat of sorption (J.mol^−1^)
C	Intercept (mg/g)
C_0_	Initial liquid-phase concentration (mg/L)
C_e_	Liquid-phase concentration of dye at equilibrium (mg/L) or Temkin constant
C_t_	Liquid-phase concentration of dye at any time t (mg/L)
d	Mean particle diameter (cm)
D	Intraparticle diffusion coefficient (cm^2^/s)
D_0_	Molecular diffusivity (cm^2^/s)
D_f_	Effective diffusion coefficient (cm^2^/s)
E_a_	Energy activation (kJ/mol)
F	Fractional approach to equilibrium, defined as $q_{t}/q_{e}$
K_1_	Equilibrium rate constant of pseudo-first-order adsorption (min^−1^)
K_2_	Pseudo-second-order rate constant of adsorption (mg/g.min)
K_bs_	Brouers–Sotolongo isotherm constant (L/mg)
k_d_	Distribution coefficient (L/g)
K_F_	Freundlich constant (L/g)
K_f_	Film diffusion constant (min^−1^)
K_H_	Hill isotherm constant (L/g)
k_in_	Intra-particle rate constant (mg min^0.5^/g)
K_L_	Langmuir constant (L/mg)
K_RP_	Redlich–Peterson model isotherm constant (L/g)
K_sp_	Solubility constant
K_T_	Toth model constant (L/mg)
M	Molecular weight of the solute (g/mol)
N	Observations number in the experimental data
n	Effective non-integer reaction order
n_F_	Freundlich equation exponent
n_H_	Hill cooperativity coefficient
n_KC_	Koble–Corrigan parameter
n_T_	Toth model exponent
q_bs_	Maximum adsorption capacity according to Brouers–Sotolongo isotherm (mg/g)
q_e_	Amount of dye adsorbed at equilibrium (mg/g)
q_e,cal_	Calculated values of adsorption uptake (mg/g)
q_e,exp_	Experimental adsorption capacity at equilibrium (mg/g)
q_e,exp_	Mean experimental and the calculated values of adsorption uptake (mg/g)
q_H_	Maximum adsorption capacity according to Hill isotherm (mg/g)
q_K_	Maximum adsorption capacity forecasted by Khan isotherm (mg/g)
q_max_	Maximum amount of the adsorbate per unit weight of the adsorbent (mg/g)
q_T_	Maximum adsorption capacity according to Toth isotherm (mg/g)
q_t_	Amount of adsorbed dye (mg/g)
q_t_	Sorbed quantity at time t (mg/g)
r	Mean radius of adsorbent particles (cm)
R	Gas constant (J/mol.k)
R^2^	Correlation coefficient
t	Contact time (min)
T	Temperature (K)
V	Solution volume (L)
W	Dry adsorbent mass (g)
Greek letters	
α	Initial sorption rate and the parameter (mg/g.min)
α_bs_	Brouers-sotolongo model exponent
α_RP_	Redlich–Peterson model constant (L/mg)^βRP^
β	Desorption constant (g/mg)
β_RP_	Redlich–Peterson model exponent
γ	Fractal time exponent
ΔG°	Gibbs energy (KJ/mol)
ΔH°	Enthalpy (KJ/mol)
ΔS°	Entropy (J/mol.K)
ρ_p_	Adsorbent density (g/cm^3^)
τ	Characteristic time (min)
τ_50%_	Half sorption time (min)

## References

[1] Chouchene A., Jeguirim M., Favre-Reguillon A., Trouvé G., Le Buzit G., Khiari B., Zagrouba F. (2012). Energetic valorisation of olive mill wastewater impregnated on low cost absorbent: Sawdust versus olive solid waste. Energy.

[2] Wakkel M., Khiari B., Zagrouba F. (2019). Textile wastewater treatment by agro-industrial waste: Equilibrium modelling, thermodynamics and mass transfer mechanisms of cationic dyes adsorption onto low-cost lignocellulosic adsorbent. J. Taiwan Inst. Chem. Eng..

[3] Singh N.B., Nagpal G., Agrawal S., Rachna (2018). Water purification by using Adsorbents: A Review. Environ. Technol. Innov..

[4] Bhatnagar A., Kaczala F., Hogland W., Marques M., Paraskeva C.A., Papadakis V.G., Sillanpää M. (2014). Valorization of solid waste products from olive oil industry as potential adsorbents for water pollution control—A review. Environ. Sci. Pollut. Res..

[5] Chouchene A., Jeguirim M., Trouvé G. (2014). Biosorption performance, combustion behavior, and leaching characteristics of olive solid waste during the removal of copper and nickel from aqueous solutions. Clean Technol. Environ. Policy.

[6] González-García P. (2018). Activated carbon from lignocellulosics precursors: A review of the synthesis methods, characterization techniques and applications. Renew. Sustain. Energy Rev..

[7] Chuah T.G., Jumasiah A., Azni I., Katayon S., Thomas Choong S.Y. (2005). Rice husk as a potentially low-cost biosorbent for heavy metal and dye removal: An overview. Desalination.

[8] Liu S., Sun Y., Wang R., Mishra S.B., Duan H., Qu H. (2018). Modification of sand with iron and copper derived from electroplating wastewater for efficient adsorption of phosphorus from aqueous solutions: A combinatorial approach for an effective waste minimization. J. Clean. Prod..

[9] Mustapha A.A., Abdu N., Jibrin J.M. (2017). Adsorption of Cadmium, Copper, Lead and Zinc on Organically Amended Soil Fractions Using the Freundlich, Langmuir and Dubinin-Raduskevich Models. Int. J. Soil Sci..

[10] Hou C., Zhao D., Zhang S., Wang Y. (2018). Highly selective adsorption of Hg(II) by the monodisperse magnetic functional chitosan nano-biosorbent. Colloid Polym. Sci..

[11] Nieto L.M., Alami S.B.D., Hodaifa G., Faur C., Rodríguez S., Giménez J.A., Ochando J. (2010). Adsorption of iron on crude olive stones. Ind. Crops Prod..

[12] Belala Z., Mechati F., Jeguirim M., Belhachemi M., Addoun F. (2014). Equilibrium modelling of copper ions biosorption by date stones and palm trees waste. Environ. Eng. Manag. J..

[13] Harikishore Kumar Reddy D., Vijayaraghavan K., Kim J.A., Yun Y.-S. (2017). Valorisation of post-sorption materials: Opportunities, strategies, and challenges. Adv. Colloid Interface Sci..

[14] Anastopoulos I., Massas I., Ehaliotis C. (2015). Use of residues and by-products of the olive-oil production chain for the removal of pollutants from environmental media: A review of batch biosorption approaches. J. Environ. Sci. Health Part A.

[15] Wakkel M., Khiari B. (2018). Basic red 2 and methyl violet adsorption by date pits: Adsorbent characterization, optimization by RSM and CCD, equilibrium and kinetic studies. Environ. Sci. Pollut. Res..

[16] Bhatnagar A., Sillanpää M. (2010). Utilization of agro-industrial and municipal waste materials as potential adsorbents for water treatment—A review. Chem. Eng. J..

[17] Vilardi G., Di Palma L., Verdone N. (2018). Heavy metals adsorption by banana peels micro-powder: Equilibrium modeling by non-linear models. Chin. J. Chem. Eng..

[18] Malkoc E., Nuhoglu Y. (2007). Potential of tea factory waste for chromium (VI) removal from aqueous solutions: Thermodynamic and kinetic studies. Separ. Purif. Technol..

[19] Haimour N.M., Emeish S. (2006). Utilization of date stones for production of activated carbon using phosphoric acid. Waste Manag..

[20] Belala Z., Jeguirim M., Belhachemi M., Addoun F., Trouvé G. (2011). Biosorption of copper from aqueous solutions by date stones and palm-trees waste. Environ. Chem. Lett..

[21] Guechi E.-K., Hamdaoui O. (2016). Evaluation of potato peel as a novel adsorbent for the removal of Cu(II) from aqueous solutions: Equilibrium, kinetic, and thermodynamic studies. Desal. Water Treat..

[22] Suárez-García F., Martínez-Alonso A., Tascón J.M.D. (2002). Pyrolysis of apple pulp: Chemical activation with phosphoric acid. J. Anal. Appl. Pyrol..

[23] Yang T., Lua A.C. (2006). Textural and chemical properties of zinc chloride activated carbons prepared from pistachio-nut shells. Mater. Chem. Phys..

[24] Anastopoulos I., Karamesouti M., Mitropoulos A.C., Kyzas G.Z. (2017). A review for coffee adsorbents. J. Mol. Liquids.

[25] Baccar R., Bouzid J., Feki M., Montiel A. (2009). Preparation of activated carbon from Tunisian olive-waste cakes and its application for adsorption of heavy metal ions. J. Hazard Mater..

[26] Abdelhadi S.O., Dosoretz C.G., Rytwo G., Gerchman Y., Azaizeh H. (2017). Production of biochar from olive mill solid waste for heavy metal removal. Bioresour. Technol..

[27] Vegliò F., Beolchini F., Prisciandaro M. (2003). Sorption of copper by olive mill residues. Water Res..

[28] Rizzi V., D’Agostino F., Fini P., Semeraro P., Cosma P. (2017). An interesting environmental friendly cleanup: The excellent potential of olive pomace for disperse blue adsorption/desorption from wastewater. Dyes Pigments.

[29] Pagnanelli F., Mainelli S., Vegliò F., Toro L. (2003). Heavy metal removal by olive pomace: Biosorbent characterisation and equilibrium modelling. Chem. Eng. Sci..

[30] Šćiban M., Klašnja M., Škrbić B. (2008). Adsorption of copper ions from water by modified agricultural by-products. Desalination.

[31] Mo K.H., Alengaram U.J., Jumaat M.Z., Yap S.P., Lee S.C. (2016). Green concrete partially comprised of farming waste residues: A review. J. Clean. Prod..

[32] Chouchene A., Jeguirim M., Khiari B., Trouvé G., Zagrouba F. (2010). Study on the emission mechanism during devolatilization/char oxidation and direct oxidation of olive solid waste in a fixed bed reactor. J. Anal. Appl. Pyrol..

[33] Martín-Lara M.A., Blázquez G., Trujillo M.C., Pérez A., Calero M. (2014). New treatment of real electroplating wastewater containing heavy metal ions by adsorption onto olive stone. J. Clean. Prod..

[34] Pagnanelli F., Mainelli S., De Angelis S., Toro L. (2005). Biosorption of protons and heavy metals onto olive pomace: Modelling of competition effects. Water Res..

[35] Alslaibi T.M., Abustan I., Ahmad M.A., Foul A.A. (2014). Kinetics and equilibrium adsorption of iron (II), lead (II), and copper (II) onto activated carbon prepared from olive stone waste. Desal. Water Treat..

[36] Fernando A., Monteiro S., Pinto F., Mendes B. (2009). Production of biosorbents from waste olive cake and its adsorption characteristics for Zn2+ ion. Sustainability.

[37] González J.F., Román S., Encinar J.M., Martínez G. (2009). Pyrolysis of various biomass residues and char utilization for the production of activated carbons. J. Anal. Appl. Pyrol..

[38] Brunauer S., Emmett P.H., Teller E. (1938). Adsorption of Gases in Multimolecular Layers. J. Am. Chem. Soc..

[39] Miraboutalebi S.M., Nikouzad S.K., Peydayesh M., Allahgholi N., Vafajoo L., McKay G. (2017). Methylene blue adsorption via maize silk powder: Kinetic, equilibrium, thermodynamic studies and residual error analysis. Process Safety Environ. Prot..

[40] Elhafez S.E.A., Hamad H.A., Zaatout A.A., Malash G.F. (2017). Management of agricultural waste for removal of heavy metals from aqueous solution: Adsorption behaviors, adsorption mechanisms, environmental protection, and techno-economic analysis. Environ. Sci. Pollut. Res..

[41] Hawari A., Khraisheh M., Al-Ghouti M.A. (2014). Characteristics of olive mill solid residue and its application in remediation of Pb^2+^, Cu^2+^ and Ni^2+^ from aqueous solution: Mechanistic study. Chem. Eng. J..

[42] Kordoghli S., Paraschiv M., Tazerout M., Khiari B., Zagrouba F. (2016). Novel Catalytic Systems for Waste Tires Pyrolysis: Optimization of Gas Fraction. J. Energy Resour. Technol..

[43] Ali R.M., Hamad H.A., Hussein M.M., Malash G.F. (2016). Potential of using green adsorbent of heavy metal removal from aqueous solutions: Adsorption kinetics, isotherm, thermodynamic, mechanism and economic analysis. Ecol. Eng..

[44] Ding T.Y., Hii S.L., Ong L. (2012). Comparison of pretreatment strategies for conversion of coconut husk fiber to fermentable sugars. BioResources.

[45] Tchoumou M., Mananga C., Bitalika C. (2015). Removal of copper (II) and nickel (II) from aqueous solution by adsorption on sugarcane bagasse. Int. J. Environ. Sci..

[46] Malkoc E., Nuhoglu Y., Dundar M. (2006). Adsorption of chromium(VI) on pomace—An olive oil industry waste: Batch and column studies. J. Hazard. Mater..

[47] Sun Z.X., Sköld R.O. (2001). A multi-parameter titration method for the determination of formation pH for metal hydroxides. Miner. Eng..

[48] Brouers F. (2014). The fractal (BSf) kinetics equation and its approximations. J. Mod. Phys..

[49] Kesraoui A., Selmi T., Seffen M., Brouers F. (2017). Influence of alternating current on the adsorption of indigo carmine. Environ. Sci. Pollut. Res. Int..

[50] Bohli T., Ouederni A., Fiol N., Villaescusa I. (2015). Evaluation of an activated carbon from olive stones used as an adsorbent for heavy metal removal from aqueous phases. C. R. Chim..

[51] Ferhat R., Laroui S., Zitouni B., Lekbir A., Abdeddaim M., Smaili N., Mohammedi Y. (2014). Experimental study of solid waste olive’s mill: Extraction modes optimization and physicochemical characterization. J. Nat. Prod. Plant Resour..

[52] Neto V.O., Oliveira A.G., Teixeira R.N., Silva M.A., Freire P.C., De Keukeleire D., Nascimento R.F. (2011). Use of coconut bagasse as alternative adsorbent for separation of copper (II) ions from aqueous solutions: Isotherms, kinetics, and thermodynamic studies. BioResources.

[53] Lasheen M.R., Ammar N.S., Ibrahim H.S. (2012). Adsorption/desorption of Cd(II), Cu(II) and Pb(II) using chemically modified orange peel: Equilibrium and kinetic studies. Solid State Sci..

[54] Zhu C.S., Wang L.P., Chen W.b. (2009). Removal of Cu(II) from aqueous solution by agricultural by-product: Peanut hull. J. Hazard. Mater..

[55] Hossain M., Ngo H.H., Guo W., Nguyen T. (2012). Removal of copper from water by adsorption onto banana peel as bioadsorbent. Int. J. Geomate.

[56] Iqbal M., Saeed A., Kalim I. (2009). Characterization of Adsorptive Capacity and Investigation of Mechanism of Cu^2+^, Ni^2+^ and Zn^2+^ Adsorption on Mango Peel Waste from Constituted Metal Solution and Genuine Electroplating Effluent. Separ. Sci. Technol..

[57] Jeguirim M., Limousy L., Labaki M., Galanakis C.M. (2017). Chapter 9—Environmental applications of coffee processing by-products. Handbook of Coffee Processing By-Products.

